# Targeting Membrane Transport and Energy Metabolism for the Identification of Repurposed Drug Candidates Against *Neisseria gonorrhoeae* Using an In Silico Strategy

**DOI:** 10.3390/antibiotics15060616

**Published:** 2026-06-17

**Authors:** Inês Pereira, André Pina, Inês Vítor, Beatriz Santos, Ana Henriques, Isabel Couto, Filomena M. Pereira, Miguel Viveiros, Ana Armada, Sofia Santos Costa, Liliana Rodrigues

**Affiliations:** Global Health and Tropical Medicine, GHTM, LA-REAL, Instituto de Higiene e Medicina Tropical, IHMT, Universidade NOVA de Lisboa, 1349-008 Lisboa, Portugal; a21002124@ihmt.unl.pt (I.P.); icouto@ihmt.unl.pt (I.C.); flmpereira@ihmt.unl.pt (F.M.P.); mviveiros@ihmt.unl.pt (M.V.); aarmada@ihmt.unl.pt (A.A.); scosta@ihmt.unl.pt (S.S.C.)

**Keywords:** *Neisseria gonorrhoeae*, antimicrobial resistance, efflux pumps, energy metabolism, drug repurposing, in silico drug repurposing

## Abstract

Background/Objectives: *Neisseria gonorrhoeae* is a high-priority pathogen for the development of new therapeutic alternatives. Efflux pumps are attractive drug targets because their inactivation influences *N. gonorrhoeae* susceptibility to multiple antimicrobials. Since most gonococcal efflux systems are energy-dependent, interference with energy metabolism and membrane transport may indirectly compromise efflux activity. Efflux inhibitors may increase intracellular antibiotic concentration, although this requires validation in resistant strains. The most effective efflux inhibitors interfere with energy metabolism, affecting several physiological processes, including efflux. In this work, we used an in silico drug repurposing strategy targeting proteins involved in membrane transport and energy metabolism in *N. gonorrhoeae*. A subset of candidate drugs were subsequently evaluated in vitro using only the reference strain *N. gonorrhoeae* ATCC 49226. Methods: Predicted drug–target interactions were identified using publicly available databases such as DrugBank and STITCH. Minimum inhibitory concentrations (MICs) of selected drugs against *N. gonorrhoeae* were determined by microdilution. Changes in intracellular ethidium bromide accumulation were assessed by real-time fluorometry as an indirect indicator of possible efflux-related interference. Results: In silico analysis identified 32 predicted targets associated with 57 approved drugs. Triclabendazole and dequalinium showed the lowest MIC values of the tested compounds (2 and 4 mg/L, respectively). Ketotifen and verapamil demonstrated activity consistent with possible efflux interference, as indicated by increased ethidium bromide accumulation. Atovaquone showed adjuvant-like effects in combination assays, suggesting that mechanisms other than efflux-related interference may contribute to its activity. Conclusions: Overall, this preliminary study identifies approved drugs with antimicrobial or adjuvant activity against a single *N. gonorrhoeae* reference strain, supporting further investigation in clinically relevant and efflux-variant strains.

## 1. Introduction

The global increase in bacterial antimicrobial resistance (AMR) represents a major threat to public health. In 2021, AMR was estimated to be directly responsible for approximately 1.14 million deaths worldwide, a number projected to reach 1.91 million annually by 2050 [1]. In response to this global challenge, the World Health Organization published a priority list of antibiotic-resistant bacteria for which new therapeutic options are urgently needed [2]. Drug-resistant *Neisseria gonorrhoeae* was classified as a high-priority pathogen.

Gonorrhoea remains one of the most common sexually transmitted infections globally, with a high incidence and considerable epidemic potential. According to the most recent European Centre for Disease Prevention and Control (ECDC) report, 96,969 confirmed cases of gonorrhoea were recorded in 2023 across the European Union and European Economic Area [3]. Similarly, provisional data from the Centers for Disease Control estimated 601,319 cases in the United States in the same year [4]. Furthermore, *N. gonorrhoeae* has exhibited rising levels of AMR, progressively limiting treatment options and reducing the likelihood of a cure. Data from the European Gonococcal Antimicrobial Surveillance Programme (Euro-GASP) revealed an increase in azithromycin resistance in *N. gonorrhoeae* of 15.9% from 2019 to 2022, alongside an 8.4% increase in ciprofloxacin resistance over the same period [5]. Although the susceptibility to extended-spectrum cephalosporins (ESCs), including ceftriaxone and cefixime, remains high, reports of decreased susceptibility and resistant isolates have increased in several countries, emphasizing the importance of continuous surveillance, treatment guideline revision and the development of new therapeutic alternatives [5,6].

Current first-line treatment primarily relies on ESCs, namely ceftriaxone or cefixime, with or without azithromycin, depending on regional guidelines. However, the emergence of multidrug-resistant (MDR) or extensively drug-resistant (XDR) *N. gonorrhoeae* strains has raised concerns regarding the potential for untreatable gonorrhoea. Second-line treatment options remain limited, and only a small number of novel anti-gonococcal agents have progressed to clinical trials [7].

Recently, zoliflodacin and gepotidacin have been approved for the treatment of urogenital gonorrhoea and represent promising additions to the therapeutic arsenal [7,8]. Nevertheless, zoliflodacin has shown lower cure rates at the oropharyngeal site, an important reservoir of infection, and gepotidacin has demonstrated reduced efficacy against fluoroquinolone-resistant strains and is currently recommended only in settings with limited treatment options due to restricted clinical safety data [7,9,10]. Moreover, the pre-clinical pipeline for new anti-gonococcal agents remains relatively sparse [7,8].

Although treatment failure rates remain relatively low, they are not negligible. A longitudinal analysis by Blouin et al. reported a treatment failure rate of 1.1% (n = 17/1593) [11]. Persistent or inadequately treated *N. gonorrhoeae* infections may lead to serious complications, including pelvic inflammatory disease, infertility, and ectopic pregnancy [12]. Therefore, it is crucial to continue anti-gonococcal drug discovery.

Antimicrobial resistance in *N. gonorrhoeae* is mediated by multiple mechanisms, including target site mutations, enzymatic drug inactivation and reduced intracellular drug accumulation. A major contributor to decreased susceptibility to ESCs and other antimicrobials is increased efflux, particularly mediated by overexpression of the MtrCDE efflux pump [13,14]. In addition, *N. gonorrhoeae* encodes other efflux pumps, including MacAB, NorM and MtrF, which are involved in the export of macrolides, fluoroquinolones, and sulfonamides, respectively [15,16,17,18]. Most gonococcal efflux systems are energy-dependent, relying on ATP hydrolysis or the proton motive force to drive substrate export [19]. Consequently, energy metabolism and membrane transport processes are functionally linked to efflux activity and intracellular drug accumulation.

Previous studies have shown that inactivation of efflux systems in resistant isolates can significantly influence antimicrobial susceptibility and, in some cases, restore clinical susceptibility [14]. These observations have positioned efflux pumps as attractive targets for anti-gonococcal drug discovery. In recent years, increasing interest has been directed toward the identification of efflux inhibitors, which may increase intracellular antibiotic concentration and improve the efficacy of existing antimicrobials when used in combination therapies [14,15,20,21]. While some efflux inhibitors may directly interact with efflux pump components, others such as thioridazine (a neuroleptic) and verapamil (an antihypertensive) have been suggested to exert indirect effects through interference with energy metabolism [22,23]. By disrupting energy production, these compounds may simultaneously affect several physiological processes, including the function of energy dependent efflux systems. Accordingly, targeting proteins involved in membrane transport and energy metabolism represents a potential strategy to compromise efflux capacity and increase intracellular antibiotic accumulation [24].

Drug repurposing has emerged as a complementary approach to conventional drug discovery, particularly when integrated with in silico screening approaches [25]. By identifying new antibacterial applications for drugs already approved for human use, this strategy benefits from existing pharmacokinetic and safety data, potentially reducing development time and cost. Computational repurposing methods have been increasingly applied to bacterial pathogens, including *N. gonorrhoeae*, to prioritise candidate compounds for experimental validation. For example, auranofin and fenamic acid analogues have shown repurposing potential against *N. gonorrhoeae* [26,27].

In this work, we employed an in silico drug repurposing strategy focused on proteins involved in membrane transport and/or energy metabolism in *N. gonorrhoeae*, followed by in vitro validation. Selected candidate compounds were subsequently evaluated in vitro using the reference strain *N. gonorrhoeae* ATCC 49226. This work provides a preliminary framework for the identification of repurposed drugs with antimicrobial or adjuvant activity that may support the development of alternative or adjunct therapeutic strategies for the management of gonococcal infections.

## 2. Results

### 2.1. In Silico Identification of Drugs Approved for Clinical Use in Humans with Potential Activity Against N. gonorrhoeae

**Figure 1:**
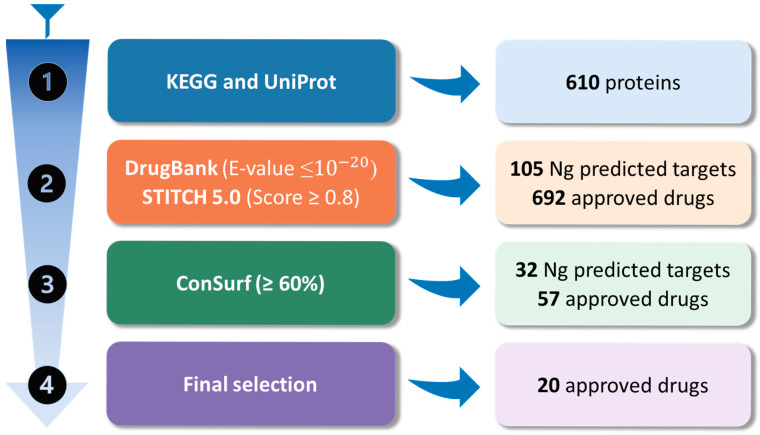
Workflow of the in silico repurposing strategy used in this study. Step 1: Searching for proteins involved with energy metabolism and membrane transport; Step 2: Sequencing similarity screenings; Step 3: Functional regions comparison; Step 4: Final selection based on previous testing, known side effects, class representation and commercial availability.

The rational workflow of the in silico strategy is summarized in Figure 1 and is inspired by a previously described methodology [21,28,29]. The in silico screening used in this study was intentionally restricted to proteins involved in membrane transport and energy metabolism. This design reflects the hypothesis that interference with these interconnected pathways may indirectly influence intracellular drug accumulation and antimicrobial susceptibility in *N. gonorrhoeae*.

A total of 610 proteins involved in energy metabolism and membrane transport, 289 from the genome of FA1090 strain and 321 from NCCP1194, was obtained from the online databases Kyoto Encyclopedia of Genes and Genomes (KEGG) and UniProt (Table S1) [30,31]. Sequence similarity screenings in the publicly available databases DrugBank and STITCH 5.0 [32,33] predicted 105 targets (55 from FA1090 and 50 from NCCP1194) associated with 692 approved drugs (Figure 1, Table S2). DrugBank and STITCH 5.0 exclusively predicted 620 (89.60%) and 24 (3.47%) of the approved drugs, respectively, whilst 48 (6.94%) drugs were predicted by both databases.

Functional regions analysis, using ConSurf Server [34], resulted in 32 predicted *N. gonorrhoeae* targets (5.2% of the interrogated targets) associated with 57 approved drugs. An example of the ConSurf analysis between an approved drug target (human carbonic anhydrase 1) and the corresponding *N. gonorrhoeae* target is presented in Figure S1.

Nineteen targets and 52 drugs were identified in the FA1090 strain, and 13 targets with 28 drugs were identified in the NCCP11945 strain (23 drugs common to both strains). Table 1 presents these targets and their potential drugs, which are approved for a variety of indications, such as the treatment of epilepsy, arrhythmia, and cancer.

The PubMed and PubChem databases were used to verify if the identified drugs had already been tested against *N. gonorrhoeae* [35]. From the 57 drugs identified using this strategy, 11 were already tested for in vitro activity against *N. gonorrhoeae*. We predicted 46 drugs that, to our knowledge, have not yet been experimentally tested against *N. gonorrhoeae* (Table 1).

**Table 1:** Predictive targets in *N. gonorrhoeae* strains FA1090 (NGO) and NCCP 11945 (NGK), with associated drugs and their corresponding therapeutic groups, identified following ConSurf analysis. References in brackets indicate previous studies, and * indicates drugs tested in this study.

Predictive Target	Drug	Group	Tested in Ng (Yes/No)
ATP synthetase (NGK_2626)	Inositol nicotinate	Vasodilator	No
Carbonic anhydrase (NGO_0574)	Amlodipine *	Antihypertensive	No
	Bendroflumethiazide	Antihypertensive	No
	Benzthiazide *	Antihypertensive	No
	Celecoxib	Anti-inflammatory	No
	Chlorothiazide	Antihypertensive	No
	Cyclothiazide	Diuretic	No
	Diazoxide	Anti-hypoglycemic	No
	Ethinamate	Anti-insomnia	No
	Hydroflumethiazide	Diuretic	No
	Methocarbamol	Muscular relaxant	No
	Quinethazone	Antihypertensive	No
	Sulpiride *	Antipsychotic	No
	Topiramate *	Anticonvulsive	No
	Trichlormethiazide	Antihypertensive	No
	Valdecoxib	Analgesic	No
Carbonic anhydrase (NGO_0574/NGK_1348)	Acetazolamide *	Diuretic	Yes [36,37]
	Brinzolamide	Antiglaucoma	No
	Chlortalidone	Antihypertensive	No
	Diclofenamide	Antiglaucoma	No
	Dorzolamide	Antiglaucoma	No
	Methazolamide	Antiglaucoma	No
	Zonisamide	Anticonvulsive	No
Carbonic anhydrase (NGK_1348)	Indapamide *	Antihypertensive	No
	Sulfanilamide	Antibiotic	Yes [38,39]
Carbonic anhydrase (NGO_0574); Phosphogluconate dehydrogenase (NGO_1914)	Furosemide	Antihypertensive	No
Cytochrome B (NGO_2030) PetB (NGK_2206)	Atovaquone *	Parasiticide	No
D-lactate dehydrogenase (NGO_0890/NGK_0915)	Hexachlorophene	Antiseptic	Yes [26]
Efflux protein (NGO_0395)	Ciprofloxacin	Antibiotic	Yes [40]
Efflux protein MtrD (NGO_1364)	Deoxycholic acid *	Bile acid	Yes [41]
	Dequalinium *	Antiseptic	Yes [42]
Enolase (NGO_0617)	Artenimol	Antimalarial	No
	Triclabendazole *	Parasiticide	Yes [43]
Flavoproteín-ubiquinone oxidoreductase (NGO_1396/NGK_1643)	Metformin *	Anti-hyperglycaemic	No
Integral membrane protein (NGO_1455/NGK_1718)	Deferoxamine *	Chelating agent	Yes [44]
MexB (NGO_1364); OprM (NGO_1363/NGK_1596)	Cefiderocol	Antibiotic	Yes [45]
NADH-quinone oxireductase (NGO_1744/NGK_2146)	Desflurane	Anaesthetic	No
NADH-quinone oxireductase (NGO1750, NGO1749, NGO1748); NADH-dehydrogenase (NGK_2150, NGK_2152)	Doxorubicin	Antineoplastic	No
NADH-quinone oxireductase (NGO_1744/NGK_2146)	Halothane	Anaesthetic	No
	Isoflurane		No
	Methoxyflurane		No
	Sevoflurane		No
Oxoglutarate dehydrogenase (NGK_0882)	Xanthinol	Vasodilator	No
Peroxiredoxin 2 (NGK_0873)	Auranofin	Antirheumatic	Yes [46,47]
Phosphogluconate dehydrogenase (NGO_1914)	Dacarbazine *	Antineoplastic	No
	Gadopentetic acid	Contrast agent	No
	Ketotifen *	Anti-histaminic	No
	Meloxicam *	Anti-inflammatory	No
	Methotrexate	Antineoplastic	Yes [48]
Pyruvate kinase (NGO_1881) Succinate-CoA ligase (NGO_0912/NGK_0888)	Acyclovir	Antiviral	No
Serine hydroxymetiltransferase (NGO_0866)	Levoleucovorin	Antineoplastic	No
Succinate dehydrogenase (NGO_0921)	Carboxin	Antifungal	No
	Thiabendazole *	Parasiticide	No
Transporter (NGO_1355/NGK_1587)	Clomipramine *	Antidepressant	No
	Desipramine *		No
	Duloxetine *		No
	Sertraline *		No

Ng: N. gonorrhoeae.

### 2.2. Minimum Inhibitory Concentrations of Selected Drugs Against N. gonorrhoeae

A group of 20 drugs (acetazolamide, amlodipine, atovaquone, benzthiazide, clomipramine, dacarbazine, deferoxamine, dequalinium, deoxycholic acid, desipramine, duloxetine, indapamide, ketotifen, meloxicam, metformin, sertraline, sulpiride, thiabendazole, topiramate, and triclabendazole) were selected for in vitro testing against *N. gonorrhoeae* ATCC^®^ 49226^TM^ (reference strain) [49]. These candidate compounds were prioritized for in vitro testing based on predicted interaction strength, target relevance and drug availability for experimental validation. This selection strategy aimed to maximize the likelihood of identifying compounds with measurable biological effects. In addition, chlorpromazine, thioridazine and verapamil were also tested for in vitro activity against *N. gonorrhoeae*. Chlorpromazine and verapamil were identified by sequence similarity screenings in DrugBank and STITCH 5.0 but were not selected after the comparison of conserved functional domains. Nevertheless, as these three compounds were previously described as efflux inhibitors in several bacterial strains [21,22,23], they were included in this study as “classical efflux inhibitors”.

All in vitro experiments were performed using the reference strain *N. gonorrhoeae* ATCC 49226. As shown in Table 2, the tested compounds presented minimum inhibitory concentration (MIC) values between 2 and >1024 mg/L, equal to the ones observed for minimum bactericidal concentrations (MBCs). Triclabendazole and dequalinium revealed the lowest MICs (2 and 4 mg/L, respectively), indicating limited antimicrobial activity under the tested conditions. The remaining compounds showed MICs values above 8 mg/L and were not considered to demonstrate relevant direct antimicrobial activity in this strain. Subsequently, these candidate compounds were further evaluated as adjuvants to the action of standard antibiotics used in the treatment of infections caused by *N. gonorrhoeae*.

### 2.3. Effect of the In Silico Selected Drugs on the MIC of Antimicrobials Used to Treat Gonorrhoea

The MICs of antimicrobials were determined in the absence and presence of the in silico selected drugs at one-fourth of their MIC value, a subinhibitory concentration selected to minimize direct effects on bacterial growth. [50]. Each compound was also tested individually in the absence of antimicrobials to confirm that this concentration did not produce detectable growth inhibition under assay conditions.

The tested antimicrobials were selected according to the categories for the treatment of gonorrhoea established by the European Centre for Disease Prevention and Control: azithromycin, cefixime and ceftriaxone (category I); ciprofloxacin, gentamicin, kanamycin and spectinomycin (category II); and tetracycline (category III) [51]. In addition, the effect of the selected drugs on the MIC of the efflux pump substrates ethidium bromide (EtBr) and tetraphenylphosphonium bromide (TPP) was also determined. Table 3 summarizes the effects of the selected compounds that promoted notable (≥four-fold) or modest (two-fold) reductions in the MICs of at least one antimicrobial or efflux pump substrate against *N. gonorrhoeae* ATCC 49226. Atovaquone showed the most pronounced activity, reducing the MIC of azithromycin and kanamycin by four- and eight-fold, respectively. Drugs such as amlodipine, clomipramine, dequalinium, desipramine, ketotifen, meloxicam, sertraline, sulpiride and verapamil promoted two-fold reductions in the MICs of several agents, including azithromycin and EtBr, suggesting a modest adjuvant-like effect. Furthermore, atovaquone, desipramine, ketotifen, sertraline and verapamil reduced the MIC of EtBr, while sertraline caused a four-fold reduction in the MIC of TPP, supporting their potential efflux inhibitory activity. These findings suggest that these compounds, particularly atovaquone and sertraline, may enhance antimicrobial activity, potentially by interfering with efflux mechanisms. None of the candidate compounds tested showed an effect on the MICs of the remaining antimicrobials, including cefixime and ceftriaxone, in the studied strain.

### 2.4. Effect of the In Silico Selected Drugs on EtBr Accumulation

**Figure 2:**
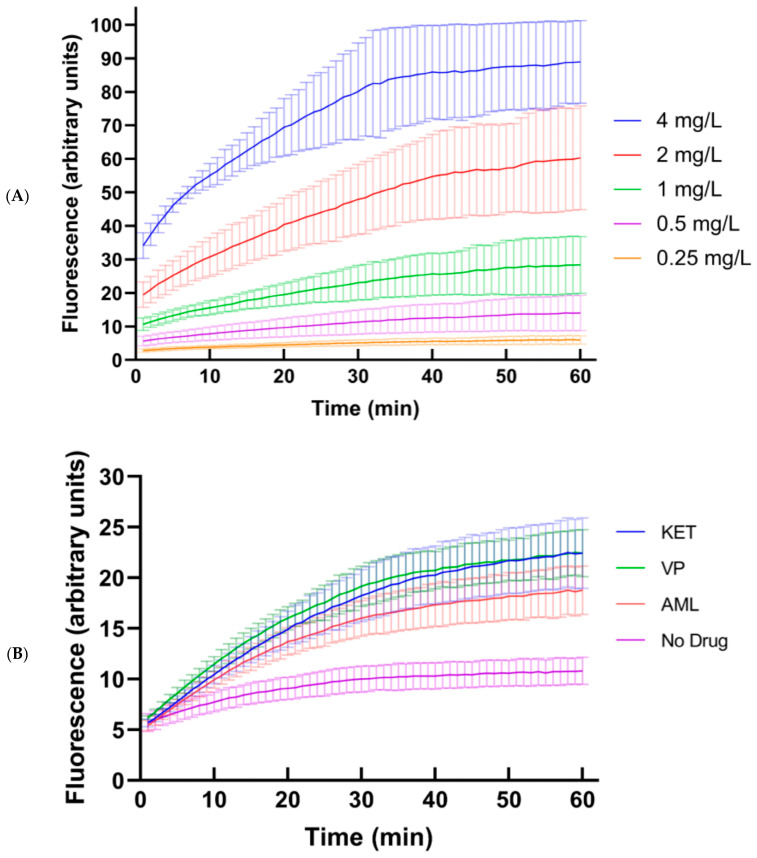
EtBr accumulation in *N. gonorrhoeae* ATCC^®^ 49226^TM^: (**A**) accumulation of increasing concentrations of EtBr (0.25–4 mg/L); (**B**) accumulation curves for the three compounds that achieved the highest levels of EtBr accumulation, namely KET, VP and AML, tested at one-fourth of their MIC. KET, ketotifen (64 mg/L); VP, verapamil (64 mg/L); AML, amlodipine (16 mg/L). Data represents the mean ± SD of three independent experiments. SD, standard deviation.

To evaluate the potential efflux-related activity of the studied drugs, EtBr accumulation assays were performed using a real-time fluorometric method, as previously described [52,53]. A concentration of 0.5 mg/L (Figure 2A) was selected for subsequent experiments, and all candidate drugs were tested at one-fourth their MICs. For clarity, Figure 2B presents only the three compounds that achieved the highest levels of EtBr accumulation in *N. gonorrhoeae* ATCC 49226, namely ketotifen, verapamil and amlodipine, together with the control condition. The complete set of accumulation curves, including all compounds that increased EtBr accumulation, is provided in Supplementary Figure S2. Ketotifen promoted the highest increase in EtBr accumulation, reaching levels comparable to those obtained for the classical inhibitor verapamil, indicating activity consistent with interference with efflux-related processes. As presented in Table 4, the relative final fluorescence (RFF) value determined for each drug showed that only ketotifen and the classical inhibitor verapamil reached RFF values ≥ 1, a result compatible with potential efflux inhibitory activity. Amlodipine showed a mild increase in EtBr accumulation but an RFF < 1. Other drugs, including chlorpromazine, thioridazine, clomipramine, desipramine, duloxetine, sertraline, dequalinium and acetazolamide, caused only minor increases in EtBr accumulation (Figure S1). The remaining drugs tested showed no effect on EtBr accumulation.

It is important to note that although increased EtBr accumulation was observed for selected compounds, these results do not allow for direct attribution to specific efflux pump inhibition but may rather reflect interference with upstream processes, including energy metabolism or membrane transport, which indirectly influence intracellular compound accumulation.

## 3. Discussion

The global burden of sexually transmitted infections, including those caused by *N. gonorrhoeae*, has been steadily increasing, primarily due to behavioural factors related to sexual practices. Current treatment for gonorrhoea relies on the use of ceftriaxone or cefixime, administered either alone or in combination with azithromycin. However, the emergence and dissemination of MDR or XDR strains has raised serious concerns about the potential threat of untreatable gonorrhoea, underscoring the urgent need for new therapeutic strategies [54]. Traditional drug discovery is a lengthy process, often requiring up to 16 years and investments of approximately 2.5 billion USD to bring a new drug to market [55]. In contrast, drug repurposing has emerged as a promising, cost-effective alternative. This strategy significantly reduces both development time (approximately six years) and associated costs (approximately 300 million USD), as it focuses on identifying new therapeutic applications for drugs already approved for clinical use in humans [56].

This study explored an in silico guided drug repurposing strategy [21,28,29] intentionally focusing on proteins involved in membrane transport and energy metabolism in *N. gonorrhoeae*, reflecting the hypothesis that interference with these pathways may indirectly affect antimicrobial susceptibility. The subsequent in vitro evaluation served as an initial biological filter, allowing for the assessment of whether computational predictions translated into observable effects under experimental conditions. However, the observed phenotypic effects cannot be considered direct confirmation of the predicted drug–target interactions. Rather, the computational pipeline was used as a prioritization tool, while in vitro assays provided preliminary evidence of biological activity in a single reference strain (*N. gonorrhoeae* ATCC 49226).

### 3.1. In Silico Selected Drugs with the Most Promising Antimicrobial Activity Against N. gonorrhoeae

Among the evaluated compounds, triclabendazole and dequalinium showed comparatively lower MIC values and were therefore considered the most active under the tested experimental conditions, supporting their prioritization for further investigation within repurposing strategies.

Dequalinium is a broad-spectrum antiseptic that acts on Gram-positive and Gram-negative bacteria, as well as yeasts and protozoa. Its mechanism of action involves increasing cell membrane permeability with subsequent loss of enzymatic activity, protein denaturation, and nucleic acid precipitation [42]. The MIC observed in our study aligns with previous findings by Chitsaz et al., who reported MIC values equal to or lower than 16 mg/L for *N. gonorrhoeae* [42]. Our in silico analysis presented MtrD, a component of the MtrCDE efflux system, as a potential target of dequalinium. Prior studies have shown that dequalinium can interact with several bacterial multidrug efflux pumps, such as AcrB, EmrE and QacR [57]. However, in our assays, dequalinium did not reduce the MIC of EtBr, nor did it demonstrate any efflux inhibitory activity in EtBr accumulation assays (RFF value of 0.26 ± 0.02). Although these findings suggest that dequalinium may not act as an efflux inhibitor in *N. gonorrhoeae* ATCC 49226, its antimicrobial activity remains noteworthy. Additional studies involving a panel of strains and clinical isolates are needed to confirm the efficacy of dequalinium and to elucidate possible multiple mechanisms of action.

Triclabendazole, an antihelmintic agent used in the treatment of fascioliasis, has an incompletely understood mechanism of action. Previous studies have proposed that it may disrupt membrane potential, interfere with tubulin dynamics, and inhibit protein synthesis [58]. In our in silico repurposing approach, enolase (NGO_0617), a key enzyme associated with carbohydrate degradation via glycolysis, was identified as a potential target in *N. gonorrhoeae*. Triclabendazole has previously demonstrated activity against *Staphylococcus aureus* and *Staphylococcus pseudintermedius*, with MICs ranging from 2 to 4 mg/L [43]. However, the same study reported MIC values above 256 mg/L for triclabendazole against *N. gonorrhoeae* ATCC 16599 and 49226 strains. This discrepancy may be attributable to differences in the methodologies employed for MIC determination across studies. Further investigation is required to clarify the antibacterial potential of triclabendazole against *N. gonorrhoeae*, as well as its mechanism of action in this species.

### 3.2. In Silico Selected Drugs as Adjuvants of Antibiotic Activity in N. gonorrhoeae

In addition to antimicrobial activity, we also evaluated the ability of each selected drug to act as an adjuvant to the activity of antimicrobials against *N. gonorrhoeae* ATCC 49226. Among the 20 in silico selected drugs, atovaquone demonstrated the most consistent adjuvant-associated effect in the tested strain. It promoted the activity of azithromycin, a category I antibiotic used in gonorrhoea treatment [51], and kanamycin. None of the other tested drugs exhibited a notable adjuvant effect for the antimicrobials analyzed in this strain. However, four drugs—desipramine, ketotifen, sertraline and verapamil—were able to slightly reduce the MIC of azithromycin (two-fold) and produced four-fold or greater reductions in the MIC of EtBr, suggesting possible interference with efflux-related processes. Notably, sertraline also reduced the MIC of tetracycline by two-fold and produced a four-fold reduction in the MIC of TPP. Although these effects were modest and observed in a single laboratory strain, they point to the potential adjuvant properties of these compounds, particularly through mechanisms that may alter intracellular compound accumulation. Further studies using clinical isolates and strains with higher efflux activity will be essential to confirm and expand upon these findings.

(a) Atovaquone

Atovaquone is a parasiticidal agent used in the treatment of malaria and pneumocystosis, acting through selective inhibition of mitochondrial cytochrome bc1 complex [59,60]. In this study, cytochrome B, which includes the bc1 complex that is essential for ATP production in *N. gonorrhoeae* [61], was identified as a predictive target. Atovaquone promoted the reduction of the MIC of azithromycin and kanamycin against *N. gonorrhoeae* ATCC 49226. However, given its known risk for of resistance development when used in monotherapy regimens [59], evaluating the resistance potential in *N. gonorrhoeae* will be crucial before further exploring the potential therapeutic applicability of this drug.

(b) Desipramine, ketotifen, sertraline and verapamil

Desipramine is a tricyclic antidepressant that inhibits norepinephrine and serotonin reuptake [62]. It presented NGO_1355, a putative transporter, as a predictive target, and reduced the MIC of EtBr and slightly decreased the MIC of azithromycin. Interestingly, this drug is known to inhibit acidic sphingomyelinase, an enzyme involved in a signalling pathway crucial for *N. gonorrhoeae* entry into host cells [62,63]. Thus, it would be interesting to continue to explore the distinct modes of action of this drug against *N. gonorrhoeae*.

Ketotifen is a histamine H1 receptor blocker used to treat allergic conditions that has been explored for potential repurposing for the treatment of toxoplasmosis and leishmaniosis [64,65,66]. In this study, ketotifen was predicted to target phosphogluconate dehydrogenase (NGO_1914), involved in the gonococcal gluconate metabolism pathway. This drug showed a slight decrease in the MIC of azithromycin and apparent efflux-related interference in the studied *N. gonorrhoeae* strain, meriting further investigation.

Sertraline, a selective serotonin reuptake inhibitor used to treat depression and manage other psychiatric disorders, presented a putative transporter (NGO_1355/NGK_1587) as a predictive target, slightly reduced the MICs of both azithromycin and tetracycline and markedly decreased the MICs of EtBr and TPP. Previous studies reported adjuvant effects when using sertraline in combination with various antibiotics in *S. aureus*, *Escherichia coli* and *Pseudomonas aeruginosa* strains [67,68]. These findings support the hypothesis that sertraline may act synergistically with antimicrobials, particularly in strains with increased efflux activity. Its potential as an adjuvant in resistant *N. gonorrhoeae* strains warrants further investigation.

Verapamil is a calcium channel blocker, used in cardiology, that has been shown to enhance the efficacy of several antibiotics by inhibiting efflux pumps in multiple pathogens, including *Mycobacterium tuberculosis* [21,22,23]. Although verapamil was initially identified by in silico screening using DrugBank and STITCH 5.0, it was not prioritized following comparative analysis of conserved functional domains. Nevertheless, verapamil was included in this study as a “classical efflux inhibitor”. Our in silico analysis revealed several ABC transporters, such as multidrug ABC transporter ATP-binding protein (NGO_1732), as potential targets of verapamil in *N. gonorrhoeae*. In vitro assays demonstrated that verapamil considerably increased EtBr accumulation (RFF ≥ 1) in *N. gonorrhoeae* ATCC 49226, indicative of efflux-related interference. Although verapamil did not greatly reduce the MICs of the tested antimicrobials, the consistent effect on EtBr transport and MIC highlights its potential as an efflux inhibitor in *N. gonorrhoeae*. Further studies are required to elucidate the mechanism of action of verapamil and its interactions with efflux systems in this pathogen.

### 3.3. In Silico Repurposed Drugs as Potential Efflux Inhibitors in N. gonorrhoeae

Efflux pumps are key contributors to antimicrobial resistance in *N. gonorrhoeae*, particularly the MtrCDE system, which actively exports a broad range of structurally diverse substrates. Inhibition of efflux pumps can enhance intracellular drug concentrations, improve or restore susceptibility in resistant strains and delay the emergence of resistance. Despite the therapeutic potential of efflux inhibitors, none have been approved for clinical application due to issues such as cytotoxicity and poor selectivity. For example, phenylalanine-arginine β-naphthylamide (PAβN), a promising broad-spectrum inhibitor of bacterial efflux pumps, was discontinued due to cytotoxicity in mammalian cells [69,70]. Nevertheless, the advantages that these compounds may provide as a therapeutic alternative justify the continuation of studies in this field.

Recent studies have focused on targeting *N. gonorrhoeae* efflux pumps, particularly the MtrCDE system. Evert et al. have rationally designed peptides to target and disrupt the activity of each of the three protein components of MtrCDE [15]. These peptides increased the susceptibility of *N. gonorrhoeae* strains to several antibiotics in a dose-dependent manner and without cytotoxicity. An extensive pharmacophore-based approach performed by Jain et al. identified inhibitors with improved pharmacological and safety profiles from the ZINC database [71]. The authors identified five hits with high binding affinity to MtrD and favourable pharmacokinetic profiles that may be used to generate new inhibitors against *N. gonorrhoeae*.

In this study, we aimed to identify approved drugs that potentially target membrane transporters and energy metabolism pathways in *N. gonorrhoeae*, evaluating their capacity as efflux inhibitors. Based on EtBr accumulation assays, ketotifen and verapamil demonstrated activity suggestive of efflux modulation in the *N. gonorrhoeae* ATCC 49226 strain. Both drugs considerably increased EtBr accumulation and reduced the MIC of EtBr, also promoting a two-fold MIC reduction for azithromycin. These findings are consistent with the previous knowledge of ketotifen’s predicted targeting of phosphogluconate dehydrogenase (NGO_1914), suggesting potential interference with metabolic pathways, and verapamil’s activity on ABC transporters, such as NGO_1732.

Amlodipine, another calcium channel blocker with inhibitory activity against human carbonic anhydrase I [72], showed a mild increase in EtBr accumulation that did not meet the threshold for classification as an efflux inhibitor (RFF < 1). The predictive target of amlodipine in *N. gonorrhoeae* was a carbonic anhydrase (NGO_0574). Nevertheless, amlodipine reduced the MIC of kanamycin, EtBr and TPP by two-fold, suggesting potential for further development of derivatives with enhanced affinity for gonococcal targets.

In contrast, atovaquone, desipramine and sertraline likely act as adjuvants of antimicrobial activity through mechanisms independent of efflux inhibition in the tested strain.

These preliminary findings highlight the need for additional, complementary assays, such as those previously established for other bacterial species [50,52,53], to confirm the EtBr accumulation results, particularly regarding the phenotypes consistent with efflux-related interference observed for ketotifen and verapamil.

Although increased EtBr accumulation was observed for selected compounds, these findings do not demonstrate direct inhibition of specific efflux pumps. The observed effects may reflect interference with upstream processes, including energy metabolism or membrane-associated transport functions, which may influence intracellular EtBr accumulation. Ongoing efforts are focused on optimizing the EtBr efflux assay protocol to broaden and deepen this analysis.

### 3.4. Study Limitations

A key limitation of this preliminary study is that several compounds exhibited high MIC values, limiting their interpretation as direct antimicrobial candidates. Therefore, their relevance should be interpreted primarily in an exploratory context, particularly in terms of prioritization and adjuvant or mechanistic effects rather than direct therapeutic application. Further studies are required to fully characterize the mechanism(s) of action of the selected drugs in *N. gonorrhoeae*, including molecular validation of predicted targets and comprehensive ex vivo and in vivo assessment of toxicity and efficacy.

Another important limitation is the restricted number of strains analyzed. All experimental data were generated using the reference strain *N. gonorrhoeae* ATCC 49226, which limits the generalizability of the findings. Given the variability in efflux pump gene expression and differences in antimicrobial resistance profiles, particularly in MDR and XDR strains, future studies should evaluate these drugs in a broad and genetically diverse collection of isolates.

Additional limitations are inherent to drug repurposing strategies. As these drugs were originally developed for indications unrelated to *N*. *gonorrhoeae* infections, their use may be constricted by drug–drug interactions, dose-limiting toxicity and unfavourable pharmacokinetic properties at the concentrations required for antibacterial or adjuvant activity. Optimization strategies, including reformulation or structural modifications, may therefore be necessary to improve safety, bioavailability and translational potential.

Importantly, the drugs identified in this study are not proposed as immediate therapeutic options. Rather, they represent experimental tools or starting points for further investigation, including studies in strains with defined resistance mechanisms and systematic assessment of pharmacological feasibility.

Additional limitations include the absence of clinical isolates, including MDR and XDR strains, as well as the lack of efflux-overexpressing control strains. Furthermore, the predicted drug–target interactions were not experimentally validated, and no cytotoxicity assessments or pharmacokinetic/pharmacodynamic (feasibility) studies were performed. Formal checkerboard assays and fractional inhibitory concentration index determinations were not conducted, precluding definitive evaluation of synergistic interactions. Finally, the ethidium bromide accumulation assay provides only an indirect indication of altered intracellular accumulation and does not constitute a direct measure of efflux pump inhibition.

Overall, this work demonstrates the potential of in silico guided drug repurposing to support the identification of approved drugs with antimicrobial or adjuvant activity against *N. gonorrhoeae*. While preliminary and limited to a reference strain, the findings provide a rationale for further targeted studies addressing efflux-related phenotypes and clinical relevance.

## 4. Materials and Methods

### 4.1. Computational Analysis/Virtual Screening

The rationale of the strategy is inspired by a methodology previously described that can be easily adapted to several targets and microorganisms [21,28,29].

(a) Compilation of potential targets involved in membrane transport or in energy metabolism in *N. gonorrhoeae*

A list of *N. gonorrhoeae* proteins involved in membrane transport or in energy metabolism was compiled using the KEGG (https://www.genome.jp/kegg, accessed on 1 October 2020) [30] and UniProt (https://www.uniprot.org, accessed on 1 October 2020) [31] databases. In the case of KEGG, the strategy was as follows: searches were conducted in the categories “transporters”, “metabolism”, “environmental information processing”, “energy metabolism” and “membrane transport”. The search results obtained with KEGG were cross-checked and supplemented using Uniprot. Searches were conducted within the categories “transporter activity”, “ATP metabolic process”, “transmembrane transport”, “generation of precursor metabolites and energy” and “antibiotic metabolic process”. This analysis was performed for *N. gonorrhoeae* strains FA 1090 [GenBank: RefSeq NC_002946] and NCCP 11945 [GenBank: RefSeq NC_011035], both of which have complete genome sequences available in KEGG and UniProt. Given that these strains have a homology of 95.2% [73], it is expected that most of the identified proteins are present in both genomes.

The ID, gene function and amino acid sequence of each selected protein were retrieved and compiled into an Excel database for subsequent analysis. This information included the target name, the respective amino acid sequence in FASTA format, and the associated biological process.

(b) Generation of an in silico library of approved drugs, with potential activity against *N. gonorrhoeae*

DrugBank uses a search strategy that results in a similarity comparison of each query (query = protein of *N. gonorrhoeae*) with all drug targets available in the databases. Only proteins with a statistical similarity value of E-value ≤ 10^−20^ were considered potential targets [28,29]. STITCH 5.0 creates a group of interactions with a given statistical confidence between drugs and targets. Only targets with a score ≥ 0.8 were selected [28,29]. In addition, only those targets associated with approved drugs, excluding the nutraceutical class, were selected in both databases.

Each of the proteins encoded by the genes identified above was used to interrogate two publicly available web-based databases that provide detailed information on drugs and their targets: DrugBank and STITCH 5.0 [32,33]. The search strategy used by DrugBank relies on the principle of homology, where each query (query = protein of *N. gonorrhoeae*) results in a comparison by similarity with all drug targets known to the database [32]. Only proteins with a statistical similarity value of E-value ≤ 10^−20^ were considered potential targets [13,19,20]. STITCH 5.0 defines a group of interactions of variable statistical confidence between drugs and targets [33]. In this case, only targets with a score ≥ 0.8 were considered for further studies [21,28,29]. From the identified homologous targets, only the ones predicted to interact with drugs approved for clinical use in humans were selected and added to the Excel database.

(c) Selective screening of identified drugs by comparison of conserved functional domains

The identified targets were subjected to further bioinformatics filtering, namely the analysis of conservation of functional regions. The functional regions of the approved drug targets and *N. gonorrhoeae* targets were compared using The ConSurf Server (https://consurf.tau.ac.il/, accessed on 1 December 2020), a bioinformatics tool that estimates the evolutionary conservation of amino acid positions in a protein based on the phylogenetic relationships between homologous sequences [34]. This procedure was used to estimate the conservation of active sites between proteins and the preservation of affinity for the predicted *N. gonorrhoeae* drugs. The parameters for the analysis were selected as previously described [29]. When conservation between functional residues was below 60%, the putative targets were excluded from further analyses. Figure S1 shows an example of the ConSurf analysis between an approved drug target (human carbonic anhydrase 1) and the corresponding *N. gonorrhoeae* target.

Finally, a list of drugs with high predicted probability of efficacy against the selected targets was generated. To assess whether these drugs had previously been tested against *N. gonorrhoeae*, searches were conducted in the PubMed and PubChem databases [35]. Drugs were classified as “tested” when any in vitro and/or in vivo studies involving *N. gonorrhoeae* were identified and as “not tested” when no such publications were found. Priority was given to drugs that had not previously been evaluated against *N. gonorrhoeae*; nevertheless, previously studied (“tested”) drugs were not excluded, as existing data could support future investigation.

From the final curated list of drugs predicted to interact with *N. gonorrhoeae* targets, 20 compounds were selected for in vitro evaluation. Selection was based on: (i) predicted drug–target associations derived from the in silico workflow (DrugBank/STITCH combined with ConSurf filtering), prioritising targets with conserved functional regions (≥60% conservation); (ii) representation across different classes and metabolic pathways related to membrane transport and energy metabolism; and (iii) practical considerations, including commercial availability and suitability for in vitro testing. Compounds associated with severe toxicity or limited experimental feasibility were excluded, unless previous reported antibacterial activity supported their inclusion for proof-of-concept evaluation. A detailed overview of the drug selection, curation and prioritization workflow is provided in Supplementary Figure S3.

### 4.2. Reagents

Acetazolamide, amlodipine, atovaquone, benzthiazide, clomipramine, deferoxamine, dequalinium, deoxycholic acid, desipramine, duloxetine, metformin, sulpiride, thiabendazole, topiramate, chlorpromazine, thioridazine, verapamil, azithromycin, cefixime, ceftriaxone, ciprofloxacin, gentamycin, kanamycin, spectinomycin and EtBr were purchased from Merck (Darmstadt, Germany). Dacarbazine, indapamide, ketotifen, TPP and triclabendazole were purchased from Tokyo Chemical Industry Co., Ltd. (TCI; Tokyo, Japan); meloxicam and sertraline were purchased from Thermo Fisher Scientific (Waltham, MA, USA). All compounds had a reported purity ≥ 98% according to the manufacturers’ specifications and were used without additional purification.

All solutions were prepared on the day of the experiment in deionized water, except for atovaquone, azithromycin, benzthiazide, cefixime, ceftriaxone, dacarbazine, deoxycholic acid, desipramine, deferoxamine, indapamide, ketotifen, meloxicam, sertraline, thiabendazole, triclabendazole and tetracycline, which were prepared in dimethyl sulfoxide (DMSO) and diluted in sterile deionized water. The maximum final concentration of DMSO in assays did not exceed 1% (*v*/*v*). Control experiments confirmed that this concentration had no effect on bacterial growth or fluorescence measurements.

### 4.3. N. gonorrhoeae Growth Conditions

The reference strain *N. gonorrhoeae* ATCC^®^ 49226^TM^ was grown at 37 °C with 5% CO_2_ in chocolate agar PolyViteX (BioMérieux, Marcy-l’Étoile, France) for 24 h. For real-time fluorometric assays, liquid cultures were prepared in GC broth (proteose peptone 15.0 g/L, corn starch 1.0 g/L, dipotassium phosphate 4.0 g/L, monopotassium phosphate 1.0 g/L, sodium chloride 5.0 g/L) supplemented with 1% (*v*/*v*) BD BBL^TM^ IsoVitaleX (BD; Franklin Lakes, NJ, USA) and incubated at 37 °C with approximately 5% CO_2_ for 24 h.

All in vitro experiments were performed using the reference strain *N. gonorrhoeae* ATCC^®^ 49226^TM^.

### 4.4. MIC and MBC Determination

MICs of the selected drugs, antimicrobials, EtBr and TPP were determined by a microdilution method, adapted from the protocol by Foester et al. [74]. Briefly, a bacterial suspension equivalent to the McFarland 1.0 standard (BioMérieux) was prepared from a 24 h *N. gonorrhoeae* culture on chocolate agar. The final inoculum was prepared by diluting the bacterial suspension 1:15 in GC broth plus 1% (*v*/*v*) IsoVitaleX. Aliquots of 0.05 mL were transferred to each well containing 0.05 mL of two-fold serial dilutions of each compound in GC broth plus 1% (*v*/*v*) IsoVitaleX. Positive (bacteria in the absence of drug) and negative (uninoculated culture media) controls were also included. After 24 h of incubation, 0.03 mL of resazurin was added to each well, followed by an additional 1 h incubation. In the presence of viable cells, resazurin (blue) is converted to resorufin (pink). The MIC was defined as the lowest drug concentration that inhibited bacterial growth. All MIC values reported correspond to individual strain-specific measurements. Aliquots from wells at and above MIC values were inoculated in drug-free GC agar to determine MBC. Each assay was performed in triplicate.

### 4.5. MIC Determination in the Presence of the In Silico Selected Drugs

To evaluate the effect of the in silico selected drugs on the MIC of antimicrobials, EtBr and TPP, MICs were redetermined in the presence of the drugs at one-fourth their MIC, a subinhibitory concentration selected to minimize direct effects on bacterial growth. Each compound was tested individually at this concentration in the absence of antimicrobials to ensure that it did not produce detectable growth inhibition under the used experimental conditions.

MICs were determined following the method described above with an additional step that consisted of incorporating each drug in the final inoculum, ensuring they were at one-fourth of their MIC when added to the microplate wells already containing two-fold serial dilutions of antimicrobials, EtBr or TPP. Incubation and interpretation of results was performed as described above. A compound was defined as a potential antimicrobial adjuvant when, in combination assays, it promoted a ≥four-fold reduction in the MIC for the tested antimicrobial compared with the antimicrobial alone. This criterion is consistent with previous studies evaluating efflux inhibitors and antimicrobial adjuvants [50].

### 4.6. Evaluation of EtBr Accumulation by Real-Time Fluorometry

The accumulation of EtBr in *N. gonorrhoeae* was assessed by real-time fluorometry as a proxy for changes in efflux activity [50,52,53]. This assay does not allow for discrimination between specific efflux pump inhibitions and other processes influencing intracellular dye accumulation.

Bacterial cultures were grown to the mid-exponential growth phase (OD_600_ of 0.6), and the cells were harvested by centrifugation, washed in phosphate-buffered saline at pH 7.4 (PBS, Merck), and resuspended in PBS supplemented with 0.4% glucose to provide an energy source. The bacterial suspension was then divided into two aliquots: one received EtBr solution, while the other served as a background control, receiving an equivalent volume of deionized water.

The EtBr “steady-state” concentration, reflecting the balance between influx and efflux, was determined using increasing concentrations of this substrate (0.25–8 mg/L; Figure 2A) prepared in PBS. For each condition, 0.2 mL microtubes were prepared containing 0.05 mL of EtBr solution at twice the final concentrations and 0.05 mL of bacterial suspension, yielding a final volume of 0.1 mL. Fluorescence emitted by EtBr was measured using a Rotor-Gene™ 3000 system (Corbett Research, Mortlake, Australia), with excitation and emission wavelengths set at 530 and 585 nm, respectively. Fluorescence data were collected over 60 cycles of 1 min, at 37 °C, to monitor EtBr accumulation in real time. The EtBr concentration that resulted in a fluorescence plateau, indicating a balance between influx and efflux, was considered the steady-state level and used in subsequent assays.

Following this determination, the selected drugs were tested for their efflux inhibitory activity in *N. gonorrhoeae* ATCC 49226. Assays were conducted using 0.1 mL reaction volumes, consisting of 0.05 mL of bacterial suspension supplemented with 0.4% glucose and 0.05 mL of either: (i) PBS, as a negative control; or (ii) a tested drug solution at a final subinhibitory concentration corresponding to one-fourth their MIC. The EtBr was used at 0.5 mg/L, the previously determined “steady-state” concentration. Control reactions containing PBS + EtBr + compound and PBS + compound were included to assess baseline fluorescence and exclude fluorescence artefacts associated with the tested compounds. Real-time fluorescence was recorded every minute for 60 min, at 37 °C, using the same excitation and emission wavelengths.

The effect of in silico selected drugs on EtBr accumulation was evaluated according to the relative final fluorescence (RFF) value, calculated by the formula: RFF = (F_plus compound_ − F_no compound_)/F_no compound_), where “F_plus compound_” and “F_no compound_” correspond to the fluorescence at the last time point (minute 60) of the accumulation curve obtained in the presence of the drug and in its absence, respectively [50]. This parameter quantifies the effect of the tested drugs on EtBr accumulation in *N. gonorrhoeae* ATCC 49226.

### 4.7. Statistical Analysis

All experiments were performed using at least three independent biological replicates. MIC and MBC determinations, as well as antimicrobial combination assays, were conducted using technical duplicates within each experiment. EtBr accumulation assays were performed in three independent experiments under identical experimental conditions.

MIC and MBC values were determined independently in each experiment, and the modal value (i.e., the value most frequently observed across independent experiments) was reported. For antimicrobial combination assays, changes in MIC values were determined by comparing the modal MIC obtained in the presence of each compound with that obtained for the corresponding control.

For the EtBr accumulation assays, fluorescence values obtained at each time point were averaged across the independent experiments, and the corresponding standard deviation (SD) was calculated. Accumulation curves are presented as mean ± SD. RFF values were calculated from the fluorescence measurements obtained at the final time point (60 min) and are reported as mean ± SD.

Given the exploratory and proof-of-concept nature of this study, the limited number of biological replicates, and the categorical endpoint generated by MIC determinations, data were analyzed descriptively. No inferential statistical tests were performed, and therefore, terms implying statistical significance were avoided throughout the manuscript. Changes in MIC values are reported descriptively as two-fold, four-fold, or greater reductions relative to the corresponding controls.

## 5. Conclusions

This study demonstrates the applicability of a hypothesis-driven, in silico guided drug repurposing strategy to support the identification of approved drugs with antimicrobial or adjuvant activity against *N. gonorrhoeae*. By focusing on proteins involved in membrane transport and energy metabolism, this approach serves as a prioritization framework rather than a discovery of novel therapeutic targets. Accordingly, we identified 20 drugs of interest for further studies, from which ketotifen and verapamil demonstrated activity consistent with possible efflux interference.

These findings are preliminary and strain-specific, as all experimental data was obtained using a single reference strain. No conclusions regarding clinical efficacy or direct efflux pump inhibitions can be drawn at this stage. Instead, the identified compounds represent experimental tools or starting points for further studies addressing resistance mechanisms, strain diversity and pharmacological feasibility.

## Figures and Tables

**Table 2:** MICs and MBCs of in silico selected drugs against *N. gonorrhoeae* ATCC^®^ 49226^TM^.

	Drug	MIC/MBC (mg/L)
In silico selected drugs	Triclabendazole	**2**
	Dequalinium	**4**
	Deferoxamine	16
	Duloxetine	16
	Sertraline	16
	Acetazolamide	16
	Clomipramine	32
	Dacarbazine	32
	Amlodipine	64
	Desipramine	64
	Atovaquone	128
	Thiabendazole	128
	Benzthiazide	256
	Indapamide	256
	Ketotifen	256
	Meloxicam	256
	Topiramate	256
	Sulpiride	1024
	Deoxycholic acid	>1024
	Metformin	>1024
Classical efflux inhibitors	Chlorpromazine	16
	Thioridazine	16
	Verapamil	256

MBC, minimum bactericidal concentration; MIC, minimum inhibitory concentration. Values in bold correspond to the compounds with lower MICs.

**Table 3:** Effect of in silico selected drugs on the MIC (mg/L) of antimicrobials against *N. gonorrhoeae* ATCC^®^ 49226^TM^.

Antimicrobial/ EP Substrate		MIC (mg/L)												
	No Drug	+ACT	+AML	+ATV	+CLO	+DAC	+DEQ	DES	+DUL	+KET	MEL	+SER	+SUL	+VP
AZM	0.25	0.25	0.13	**0.06**	0.13	0.25	0.13	0.13	0.25	0.13	0.13	0.13	0.13	0.13
GM	1	1	1	1	1	0.5	1	1	1	1	0.5	1	0.5	1
KM	4	2	4	**0.5**	2	4	2	4	4	4	4	4	4	4
TET	0.5	0.5	0.5	0.5	0.5	0.5	0.5	0.5	0.5	0.5	0.5	0.25	0.5	0.5
EtBr	4	2	2	**1**	2	2	4	**1**	2	**1**	2	**1**	2	**1**
TPP	64	32	32	32	32	64	32	32	64	32	64	**16**	64	32

In silico selected drugs were tested at one-fourth of their MIC. Values in bold correspond to at least four-fold MIC reduction. ACT, acetazolamide; AML, amlodipine; ATV, atovaquone; AZM, azithromycin; CLO, clomipramine; DAC, dacarbazine; DEQ, dequalinium; DES, desipramine; DUL, duloxetine; EP, efflux pump; EtBr, ethidium bromide; GM, gentamicin; KET, ketotifen; KM, kanamycin; MEL, meloxicam; SER, sertraline; SUL, sulpiride; TET, tetracycline; TPP, tetraphenylphosphonium bromide; VP, verapamil.

**Table 4:** RFF values of the tested drugs in *N. gonorrhoeae* ATCC^®^ 49226^TM^.

	Drug	RFF ± SD
In silico selected drugs	**Ketotifen**	**1.13 ± 0.05**
	Amlodipine	0.81 ± 0.29
	Clomipramine	0.481 ± 0.13
	Desipramine	0.36 ± 0.17
	Duloxetine	0.34 ± 0.07
	Sertraline	0.28 ± 0.10
	Dequalinium	0.26 ± 0.02
	Acetazolamide	0.24 ± 0.02
	Triclabendazole	0.002 ± 0.03
	Sulpiride	−0.002 ± 0.10
	Indapamide	−0.03 ± 0.10
	Thiabendazole	−0.03 ± 0.16
	Atovaquone	−0.04 ± 0.05
	Meloxicam	−0.04 ± 0.04
	Topiramate	−0.05 ± 0.08
	Dacarbazine	−0.05 ± 0.15
	Deferoxamine	−0.06 ± 0.09
	Metformin	−0.13 ± 0.02
	Benzthiazide	−0.14 ± 0.08
	Deoxycholic acid	−0.24 ± 0.09
Classical efflux inhibitors	**Verapamil**	**1.14 ± 0.16**
	Chlorpromazine	0.57 ± 0.16
	Thioridazine	0.38 ± 0.16

Drugs were tested at one-fourth of their MIC values. RFF values correspond to the last fluorescence reading point at 60 min. Data represents mean ± standard deviation of three independent experiments. RFF, relative final fluorescence; SD, standard deviation. In bold are the candidate drugs with RFF ≥ 1.

## Data Availability

Data are contained within the article and Supplementary Materials.

## Supplementary Materials

The following supporting information can be downloaded at: https://www.mdpi.com/article/10.3390/antibiotics15060616/s1, Figure S1: Example of a comparison of the functional regions between an approved drug target (human carbonic anhydrase 1) and the corresponding *N. gonorrhoeae* (Ng) target (NGO_0574). A: ConSurf analysis of the approved target. B: Sequence alignment and comparison. Green boxes highlight identical functional residues. Red boxes indicate divergent residues within key functional regions; Figure S2: Complete EtBr accumulation dataset in *N. gonorrhoeae* ATCC 49226; Figure S3: Flowchart illustrating the identification, curation, filtering and prioritization process used to generate the final panel of approved drugs for in vitro testing against *N. gonorrhoeae*; Table S1: List of *N. gonorrhoeae* proteins involved in energy metabolism and membrane transport; Table S2: List of potential candidate drugs and their predicted targets in *N. gonorrhoeae* FA1090 (A) and NCCP11945 (B).

## References

[1] Naghavi M., Vollset S.E., Ikuta K.S., Swetschinski L.R., Gray A.P., Wool E.E., Aguilar G.R., Mestrovic T., Smith G., Han C. (2024). Global Burden of Bacterial Antimicrobial Resistance 1990–2021: A Systematic Analysis with Forecasts to 2050. Lancet.

[2] Tacconelli E., Carrara E., Savoldi A., Harbarth S., Mendelson M., Monnet D.L., Pulcini C., Kahlmeter G., Kluytmans J., Carmeli Y. (2018). Discovery, Research, and Development of New Antibiotics: The WHO Priority List of Antibiotic-Resistant Bacteria and Tuberculosis. Lancet Infect. Dis..

[3] European Centre for Disease Prevention and Control (2025). Gonorrhoea-Annual Epidemiological Report for 2023 [Internet]. Stockholm; Report. https://www.ecdc.europa.eu/en/publications-data/gonorrhoea-annual-epidemiological-report-2023.

[4] Centers for Disease Control and Prevention (2025). Sexually Transmitted Infections Surveillance, 2024 (Provisional)|STI Statistics|CDC [Internet]. Report. https://www.cdc.gov/sti-statistics/annual/index.html.

[5] Jacobsson S., Cole M.J., Schröder D., Jansen van Rensburg M., Day M., Ködmön C., Unemo M., Pleininger S., Schindler S., El-Khatib Z. (2025). Antimicrobial Resistance in *Neisseria gonorrhoeae* and Its Risk Groups in 23 European Countries in 2022 Within the European Gonococcal Antimicrobial Surveillance Programme (Euro-GASP): A Retrospective Observational Study. Lancet Reg. Health Eur..

[6] European Centre for Disease Prevention and Control (ECDC) (2024). Gonococcal Antimicrobial Susceptibility Surveillance in the EU/EEA.

[7] Lewis D.A. (2019). New Treatment Options for *Neisseria gonorrhoeae* in the Era of Emerging Antimicrobial Resistance. Sex. Health.

[8] Hiruy H., Bala S., Byrne J.M., Roche K.G., Jang S.H., Kim P., Nambiar S., Rubin D., Yasinskaya Y., Bachmann L.H. (2026). US Food and Drug Administration, Centers for Disease Control and Prevention, and National Institutes of Health Co-Sponsored Public Workshop Summary—Development Considerations of Antimicrobial Drugs for the Treatment of Gonorrhea. Clin. Infect. Dis..

[9] Luckey A., Balasegaram M., Barbee L.A., Batteiger T.A., Broadhurst H., Cohen S.E., Delany-Moretlwe S., Vries H.J.C., Dionne J.A., Gill K. (2026). Zoliflodacin Versus Ceftriaxone Plus Azithromycin for Treatment of Uncomplicated Urogenital Gonorrhoea: An International, Randomised, Controlled, Open-Label, Phase 3, Non-Inferiority Clinical Trial. Lancet.

[10] Ross J.D.C., Wilson J., Workowski K.A., Taylor S.N., Lewis D.A., Gatsi S., Flight W., Scangarella-Oman N.E., Jakielaszek C., Lythgoe D. (2025). Oral Gepotidacin for the Treatment of Uncomplicated Urogenital Gonorrhoea (EAGLE-1): A Phase 3 Randomised, Open-Label, Non-Inferiority, Multicentre Study. Lancet.

[11] Blouin K., Lefebvre B., Trudelle A., Defay F., Perrault-Sullivan G., Gnimatin J.P., Labbé A. (2024). *Neisseria gonorrhoeae* Treatment Failure to the Recommended Antibiotic Regimen-Québec, Canada, 2015-19. J. Antimicrob. Chemother..

[12] Allan-Blitz L.T., Fifer H., Klausner J.D. (2024). Managing Treatment Failure in *Neisseria gonorrhoeae* Infection: Current Guidelines and Future Directions. Lancet Infect. Dis..

[13] Golparian D., Shafer W.M., Ohnishi M., Unemo M. (2014). Importance of Multidrug Efflux Pumps in the Antimicrobial Resistance Property of Clinical Multidrug-Resistant Isolates of *Neisseria gonorrhoeae*. Antimicrob. Agents Chemother..

[14] Chen S., Connolly K.L., Rouquette-Loughlin C., Andrea A.D., Jerse A.E., Shafer W.M. (2019). Could Dampening Expression of the *Neisseria gonorrhoeae mtrCDE*-Encoded Efflux Pump Be a Strategy To Preserve Currently or Resurrect Formerly Used Antibiotics To Treat Gonorrhea?. mBio.

[15] Evert B.J., Slesarenko V.A., Punnasseril J.M.J., Taha, Zhan J., Zhou Y., Semchenko E.A., Seib K.L. (2022). Self-Inhibitory Peptides Targeting the *Neisseria gonorrhoeae* MtrCDE Efflux Pump Increase Antibiotic Susceptibility. Antimicrob. Agents Chemother..

[16] Rouquette-Loughlin C.E., Balthazar J.T., Shafer W.M. (2005). Characterization of the MacA-MacB Efflux System in *Neisseria gonorrhoeae*. J. Antimicrob. Chemother..

[17] Rouquette-Loughlin C., Dunham S.A., Kuhn M., Balthazar J.T., Shafer W.M. (2003). The NorM Efflux Pump of *Neisseria gonorrhoeae* and *Neisseria meningitidis* Recognizes Antimicrobial Cationic Compounds. J. Bacteriol..

[18] Su C.C., Bolla J.R., Kumar N., Radhakrishnan A., Long F., Delmar J.A., Chou T., Rajashankar K.R., Shafer W.M., Yu E.W. (2015). Structure and Function of *Neisseria gonorrhoeae* MtrF Iluminates a Class of Antimetabolite Efflux Pumps. Cell Rep..

[19] Shafer W.M., Yu E.W., Rouquette-Loughlin C., Golparian D., Jerse A.E., Unemo M., Li X.Z., Elkins C., Zgurskaya H. (2016). Efflux Pumps in *Neisseria gonorrhoeae*: Contributions to Antimicrobial Resistance and Virulence. Efflux-Mediated Antimicrobial Resistance in Bacteria.

[20] Marshall R.L., Lloyd G.S., Lawler A.J., Element S.J., Kaur J., Ciusa M.L., Ricci V., Tschumi A., Kühne H., Alderwick L.J. (2020). New Multidrug Efflux Inhibitors for Gram-Negative Bacteria. mBio.

[21] Rodrigues L., Cravo P., Viveiros M. (2020). Efflux Pump Inhibitors as a Promising Adjunct Therapy Against Drug Resistant Tuberculosis: A New Strategy to Revisit Mycobacterial Targets and Repurpose Old Drugs. Expert Rev. Anti. Infect. Ther..

[22] Weinstein E.A., Yano T., Li L.S., Avarbock D., Avarbock A., Helm D., McColm A.A., Duncan K., Lonsdale J.T., Rubin H. (2005). Inhibitors of Type II NADH:Menaquinone Oxidoreductase Represent a Class of Antitubercular Drugs. Proc. Natl. Acad. Sci. USA.

[23] Chen C., Gardete S., Jansen R.S., Shetty A., Dick T., Rhee K.Y., Dartois V. (2018). Verapamil Targets Membrane Energetics in *Mycobacterium tuberculosis*. Antimicrob. Agents Chemother..

[24] Li X.Z., Plésiat P., Nikaido H. (2015). The Challenge of Efflux-Mediated Antibiotic Resistance in Gram-negative Bacteria. Clin. Microbiol. Rev..

[25] Jourdan J.P., Bureau R., Rochais C., Dallemagne P. (2020). Drug Repositioning: A Brief Overview. J. Pharm. Pharmacol..

[26] Foerster S., Gustafsson T.N., Brochado A.R., Desilvestro V., Typas A., Unemo M. (2020). The First Wide-Scale Drug Repurposing Screen Using the Prestwick Chemical Library (1200 Bioactive Molecules) Against *Neisseria gonorrhoeae* Identifies High In Vitro Activity of Auranofin and Many Additional Drugs. APMIS.

[27] Seong Y.J., Alhashimi M., Mayhoub A., Mohammad H., Seleem M.N. (2020). Repurposing Fenamic Acid Drugs To Combat Multidrug-Resistant *Neisseria gonorrhoeae*. Antimicrob. Agents Chemother..

[28] Bispo N.A., Culleton R., Silva L.A., Cravo P. (2013). A Systematic In Silico Search for Target Similarity Identifies Several Approved Drugs with Potential Activity against the *Plasmodium falciparum* Apicoplast. PLoS ONE.

[29] Neves B.J., Braga R.C., Bezerra J.C.B., Cravo P.V.L., Andrade C.H. (2015). In Silico Repositioning-Chemogenomics Strategy Identifies New Drugs with Potential Activity against Multiple Life Stages of *Schistosoma mansoni*. PLoS Negl. Trop. Dis..

[30] Kanehisa M., Goto S. (2000). KEGG: Kyoto Encyclopedia of Genes and Genomes. Nucleic Acids Res..

[31] Bateman A., Martin M.J., Orchard S., Magrane M., Ahmad S., Alpi E., Bowler-Barnett E.H., Britto R., Bye-A-Jee H., Cukura A. (2023). UniProt: The Universal Protein Knowledgebase in 2023. Nucleic Acids Res..

[32] Wishart D.S., Feunang Y.D., Guo A.C., Lo E.J., Marcu A., Grant J.R., Sajed T., Johnson D., Li C., Sayeeda Z. (2018). DrugBank 5.0: A Major Update to the DrugBank Database for 2018. Nucleic Acids Res..

[33] Szklarczyk D., Santos A., Von Mering C., Jensen L.J., Bork P., Kuhn M. (2016). STITCH 5: Augmenting Protein-Chemical Iteraction Networks with Tissue and Affinity Data. Nucleic Acids Res..

[34] Ashkenazy H., Abadi S., Martz E., Chay O., Mayrose I., Pupko T., Ben-Tal N. (2016). ConSurf 2016: An Improved Methodology to Estimate and Visualize Evolutionary Conservation in Macromolecules. Nucleic Acids Res..

[35] Sayers E.W., Bolton E.E., Brister J.R., Canese K., Chan J., Comeau D.C., Farrell C.M., Feldgarden M., Fine A.M., Funk K. (2023). Database Resources of the National Center for Biotechnology Information in 2023. Nucleic Acids Res..

[36] Hewitt C.S., Abutaleb N.S., Elhassanny A.E.M., Nocentini A., Cao X., Amos D.P., Youse M.S., Holly K.J., Marapaka A.K., An W. (2021). Structure-Activity Relationship Studies of Acetazolamide-Based Carbonic Anhydrase Inhibitors with Activity against *Neisseria gonorrhoeae*. ACS Infect. Dis..

[37] Abutaleb N.S., Elhassanny A.E.M., Seleem M.N. (2022). In Vivo Efficacy of Acetazolamide in a Mouse Model of *Neisseria gonorrhoeae* Infection. Microb. Pathog..

[38] Cokkinis A.J., Mcelligott G.L.M. (1938). Sulphanilamide in gonorrhoea: An analysis of 633 cases. Lancet.

[39] Kampmeier R.H. (1983). Introduction of Sulfonamide Therapy for Gonorrhea. Sex. Transm. Dis..

[40] Loo P.S., Ridgway G.L., Oriel J.D. (1985). Single Dose Ciprofloxacin for Treating Gonococcal Infections in Men. Genitourin. Med..

[41] Palace S.G., Fryling K.E., Li Y., Wentworth A.J., Traverso G., Grad Y.H. (2021). Identification of Bile Acid and Fatty Acid Species as Candidate Rapidly Bactericidal Agents for Topical Treatment of Gonorrhoea. J. Antimicrob. Chemother..

[42] Chitsaz M., Booth L., Blyth M.T., O’mara M.L., Brown M.H. (2019). Multidrug Resistance in *Neisseria gonorrhoeae*: Identification of Functionally Important Residues in the MtrD Efflux Protein. mBio.

[43] Pi H., Ogunniyi A.D., Savaliya B., Nguyen H.T., Page S.W., Lacey E., Venter H., Trott D.J. (2021). Repurposing of the Fasciolicide Triclabendazole to Treat Infections Caused by *Staphylococcus* spp. and Vancomycin-Resistant Enterococci. Microorganisms.

[44] Lowy F.D., Pollack S., Fadl-Allah N., Steigbigel N.H. (1984). Susceptibilities of Bacterial and Fungal Urinary Tract Isolates to Desferrioxamine. Antimicrob. Agents Chemother..

[45] Ito A., Sato T., Ota M., Takemura M., Nishikawa T., Toba S., Kohira N., Miyagawa S., Ishibashi N., Matsumoto S. (2017). In Vitro Antibacterial Properties of Cefiderocol, a Novel Siderophore Cephalosporin, against Gram-Negative Bacteria. Antimicrob. Agents Chemother..

[46] Elkashif A., Seleem M.N. (2020). Investigation of Auranofin and Gold-Containing Analogues Antibacterial Activity Against Mltidrug-Resistant *Neisseria gonorrhoeae*. Sci. Rep..

[47] Elhassanny A.E.M., Abutaleb N.S., Seleem M.N. (2022). Auranofin Exerts Antibacterial Activity Against *Neisseria gonorrhoeae* in a Female Mouse Model of Genital Tract Infection. PLoS ONE.

[48] Baccanari D.P., Tansik R.L. (1984). Kinetics of Methotrexate Binding to Dihydrofolate Reductase from *Neisseria gonorrhoeae*. Biochem. Pharmacol..

[49] Clinical and Laboratory Standards Institute (CLSI) (2023). Performance Standards for Antimicrobial Susceptibility Testing.

[50] Costa S.S., Lopes E., Azzali E., Machado D., Coelho T., Da Silva P.E.A., Viveiros M., Pieroni M., Couto I. (2016). An Experimental Model for the Rapid Screening of Compounds with Potential Use Against Mycobacteria. Assay Drug Dev. Technol..

[51] European Centre for Disease Prevention and Control (2019). Response Plan to Control and Manage the Threat of Multi-and Extensively Drug-Resistant Gonorrhoea in Europe-2019 Update [Internet]. Stockholm; Report. https://www.ecdc.europa.eu/en/publications-data/response-plan-control-and-manage-threat-multi-and-extensively-drug-resistant-0.

[52] Paixão L., Rodrigues L., Couto I., Martins M., Fernandes P., de Carvalho C.C.C.R., Monteiro G.A., Sansonetty F., Amaral L., Viveiros M. (2009). Fluorometric Determination of Ethidium Bromide Efflux Kinetics in *Escherichia coli*. J. Biol. Eng..

[53] Rodrigues L., Viveiros M., Aínsa J.A. (2021). Measuring Efflux and Permeability in Mycobacteria. Methods Mol. Biol..

[54] Unemo M., Seifert H.S., Hook E.W., Hawkes S., Ndowa F., Dillon J.A.R. (2019). Gonorrhoea. Nat. Rev. Dis. Primers.

[55] Mohs R.C., Greig N.H. (2017). Drug Discovery and Development: Role of Basic Biological Research. Alzheimers Dement..

[56] Rao V.S., Srinivas K. (2011). Modern Drug Discovery Process: An In Silico Approach. J. Bioinform. Seq. Anal..

[57] Bailly C. (2021). Medicinal Applications and Molecular Targets of Dequalinium Chloride. Biochem. Pharmacol..

[58] El-Tantawy W.H., Salem H.F., Mohammed Safwat N.A.S. (2007). Effect of Fascioliasis on the Pharmacokinetic Parameters of Triclabendazole in Human Subjects. Pharm. World Sci..

[59] Mather M.W., Darrouzet E., Valkova-Valchanova M., Cooley J.W., McIntosht M.T., Daldal F., Vaidya A.B. (2005). Uncovering the Molecular Mode of Action of the Antimalarial Drug Atovaquone Using a Bacterial System. J. Biol. Chem..

[60] Fisher N., Meunier B., Biagini G.A. (2020). The Cytochrome bc1 Complex as an Antipathogenic Target. FEBS Lett..

[61] Li Y., Hopper A., Overton T., Squire D.J.P., Cole J., Tovell N. (2010). Organization of the Electron Transfer Chain to Oxygen in the Obligate Human Pathogen *Neisseria gonorrhoeae*: Roles for Cytochromes c4 and c5, But Not Cytochrome c2, in Oxygen Reduction. J. Bacteriol..

[62] Kornhuber J., Tripal P., Reichel M., Mühle C., Rhein C., Muehlbacher M., Groemer T.W., Gulbins E. (2010). Functional Inhibitors of Acid Sphingomyelinase (FIASMAs): A Novel Pharmacological Group of Drugs with Broad Clinical applications. Cell. Physiol. Biochem..

[63] Grassmé H., Gulbins E., Brenner B., Ferlinz K., Sandhoff K., Harzer K., Lang F., Meyer T.F. (1997). Acidic Sphingomyelinase Mediates Entry of *N. gonorrhoeae* into Nonphagocytic Cells. Cell.

[64] Sokol K.C., Amar N.K., Starkey J., Grant J.A. (2013). Ketotifen in the Management of Chronic Urticaria: Resurrection of an Od Drug. Ann. Allergy Asthma Immunol..

[65] Rezaei F., Ebrahimzadeh M.A., Daryani A., Sharif M., Ahmadpour E., Sarvi S. (2016). The Inhibitory Effect of Cromolyn Sodium and Ketotifen on *Toxoplasma gondii* Entrance Into Host Cells In Vitro and In Vivo. J. Parasit. Dis..

[66] Sarouey L.A., Khanaliha K., Rahimi-Moghaddam P., Khorrami S., Dayer M.S., Tabatabaie F. (2019). In Vitro Effects of Ketotifen and Cromolyn Sodium on Promastigotes and Amastigotes of *Leishmania major*. Jundishapur J. Microbiol..

[67] Endo T.H., Santos M.H.d.M., Scandorieiro S., Gonçalves B.C., Vespero E.C., Perugini M.R.E., Pavanelli W.R., Nakazato G., Kobayashi R.K.T. (2025). Selective Serotonin Reuptake Inhibitors: Antimicrobial Activity Against ESKAPEE Bacteria and Mechanisms of Action. Antibiotics.

[68] Ayaz M., Subhan F., Ahmed J., Khan A.-U., Ullah F., Ullah I., Ali G., Syed N., Hussain S. (2015). Sertraline Enhances the Activity of Antimicrobial Agents Against Pathogens of Clinical Relevance. J. Biol. Res..

[69] Sharma A., Gupta V.K., Pathania R. (2019). Efflux Pump Inhibitors for Bacterial Pathogens: From Bench to Bedside. Indian J. Med. Res..

[70] Lomovskaya O., Warren M.S., Lee A., Galazzo J., Fronko R., Lee M., Blais J., Cho D., Chamberland S., Renau T. (2001). Identification and Characterization of Inhibitors of Multidrug Resistance Efflux Pumps in *Pseudomonas aeruginosa*: Novel Agents for Combination Therapy. Antimicrob. Agents Chemother..

[71] Jain N., Sk M.F., Mishra A., Kar P., Kumar A. (2022). Identification of Novel Efflux Pump Inhibitors for *Neisseria gonorrhoeae* Via Multiple Ligand-Based Pharmacophores, E-pharmacophore, Molecular Docking, Density Functional Theory, and Molecular Dynamics Approaches. Comput. Biol. Chem..

[72] Puscas I., Gilau L., Coltau M., Pasca R., Domuta G., Baican M., Hecht A. (2000). Hypotensive Effect of Calcium Channel Blockers is Parallel with Carbonic Anhydrase I Inhibition. Clin. Pharmacol. Ther..

[73] Gyung T.C., Jeong S.Y., Hee B.O., Yeong S.L., Sun H.C., Sang J.K., Yoo C.K. (2008). Complete Genome Sequence of *Neisseria gonorrhoeae* NCCP11945. J. Bacteriol..

[74] Foerster S., Desilvestro V., Hathaway L.J., Althaus C.L., Unemo M. (2017). A New Rapid Resazurin-Based Microdilution Assay for Antimicrobial Susceptibility Testing of *Neisseria gonorrhoeae*. J. Antimicrob. Chemother..

