# Supplementary material for: Targeting Membrane Transport and Energy Metabolism for the Identification of Repurposed Drug Candidates Against Neisseria gonorrhoeae Using an In Silico Strategy
Source: Antibiotics (Basel). 2026 Jun 17;15(6):616. doi: 10.3390/antibiotics15060616 (PMC13295694; doi:10.3390/antibiotics15060616)
Supplement: Supplementary file 1 [file antibiotics-15-00616-s001.zip › Figure S1.pdf]

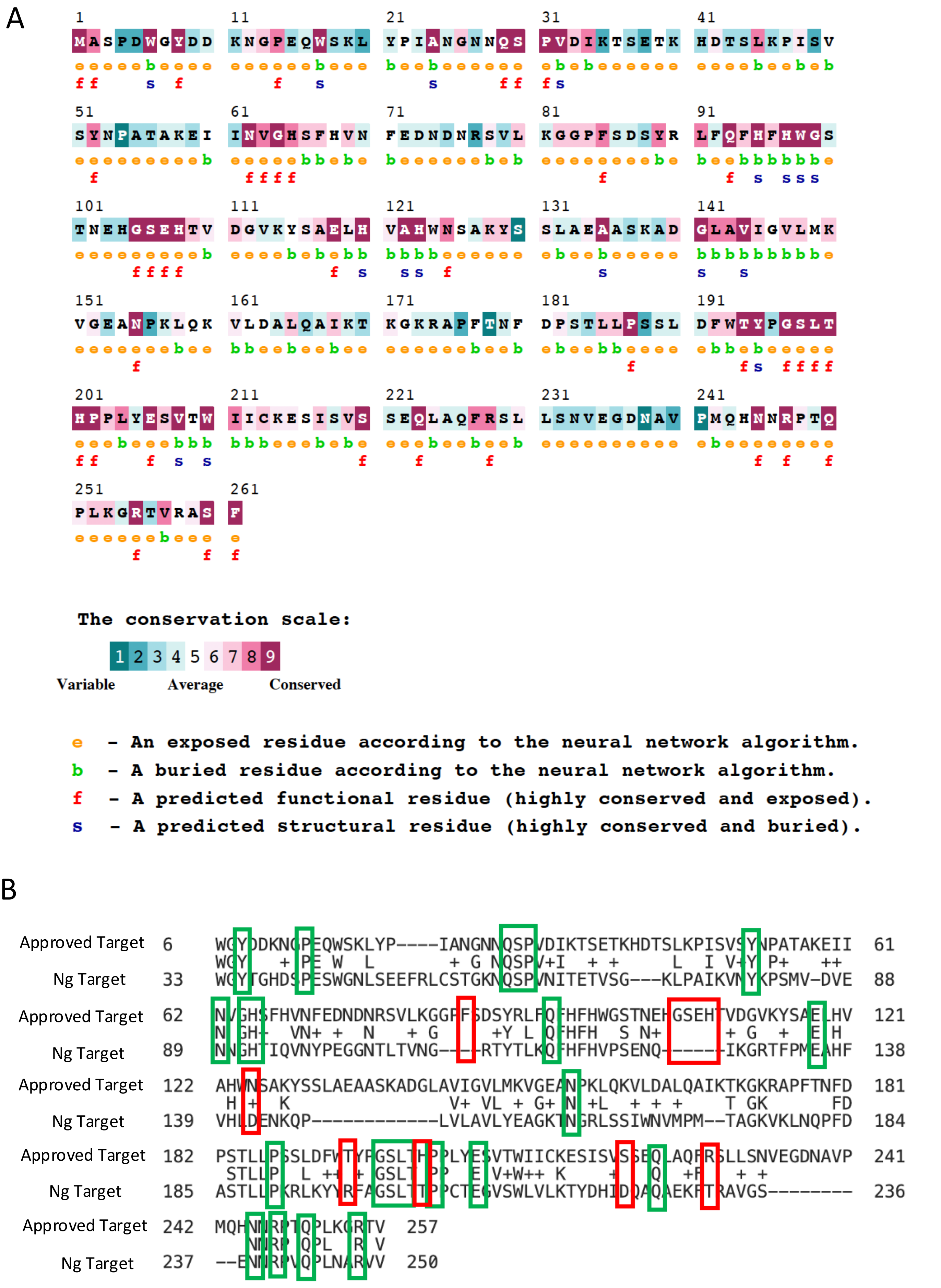

Figure S1. Example of a comparison of the functional regions between an approved drug target (human cartbonic anhydrase 1) and the corresponding *N. gonorrhoeae* (Ng) target (NGO_0574). A: ConSurf analysis of the approved target; B: Sequences alignment and comparison. Green boxes highlight identical functional residues. Red boxes indicate divergent residues within key functional regions.
