# Supplementary material for: Targeting Membrane Transport and Energy Metabolism for the Identification of Repurposed Drug Candidates Against Neisseria gonorrhoeae Using an In Silico Strategy
Source: Antibiotics (Basel). 2026 Jun 17;15(6):616. doi: 10.3390/antibiotics15060616 (PMC13295694; doi:10.3390/antibiotics15060616)
Supplement: Supplementary file 1 [file antibiotics-15-00616-s001.zip › Figure S2.pdf]

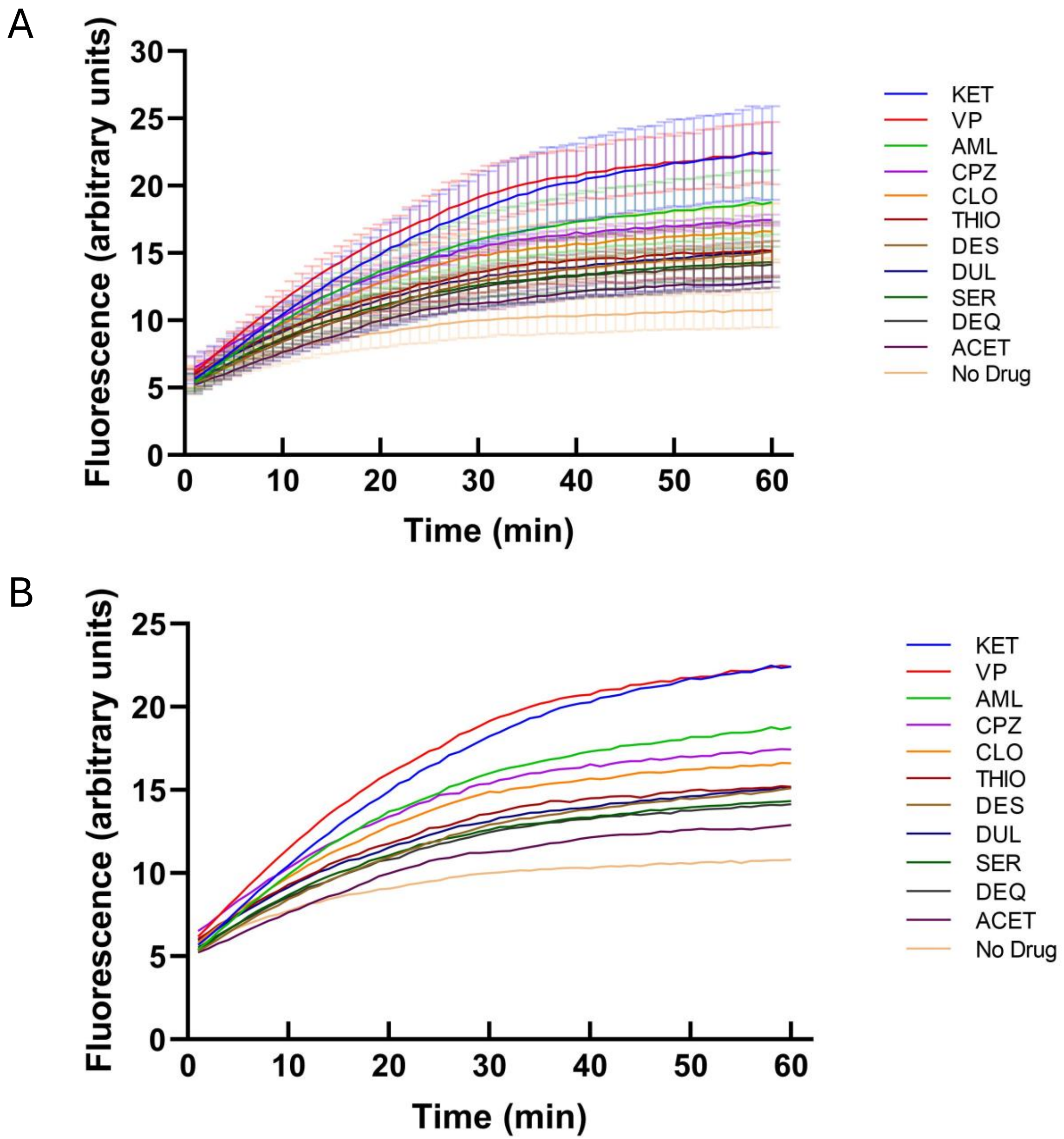

Figure S2. Complete ethidium bromide (EtBr) accumulation profiles obtained for all tested drugs in *N. gonorrhoeae* ATCC 49226. Fluorescence was monitored over 60 min in the presence of EtBr (0.5 mg/L) and each drug at ¼ MIC. Curves represent mean values from three independent experiments. To facilitate visualisation of the compounds showing the most pronounced effects on EtBr accumulation, the main manuscript (Figure 2) presents only the control and the three compounds that produced the highest EtBr accumulation, together with the corresponding error bars (mean ± SD). The complete dataset is provided here, including **(A)** accumulation curves with error bars (mean ± SD) and **(B)** the same curves displayed without error bars to improve visualisation of overlapping profiles and compounds with modest effects on EtBr accumulation.
