# Supplementary material for: Targeting Membrane Transport and Energy Metabolism for the Identification of Repurposed Drug Candidates Against Neisseria gonorrhoeae Using an In Silico Strategy
Source: Antibiotics (Basel). 2026 Jun 17;15(6):616. doi: 10.3390/antibiotics15060616 (PMC13295694; doi:10.3390/antibiotics15060616)
Supplement: Supplementary file 1 [file antibiotics-15-00616-s001.zip › Figure S3.pdf]

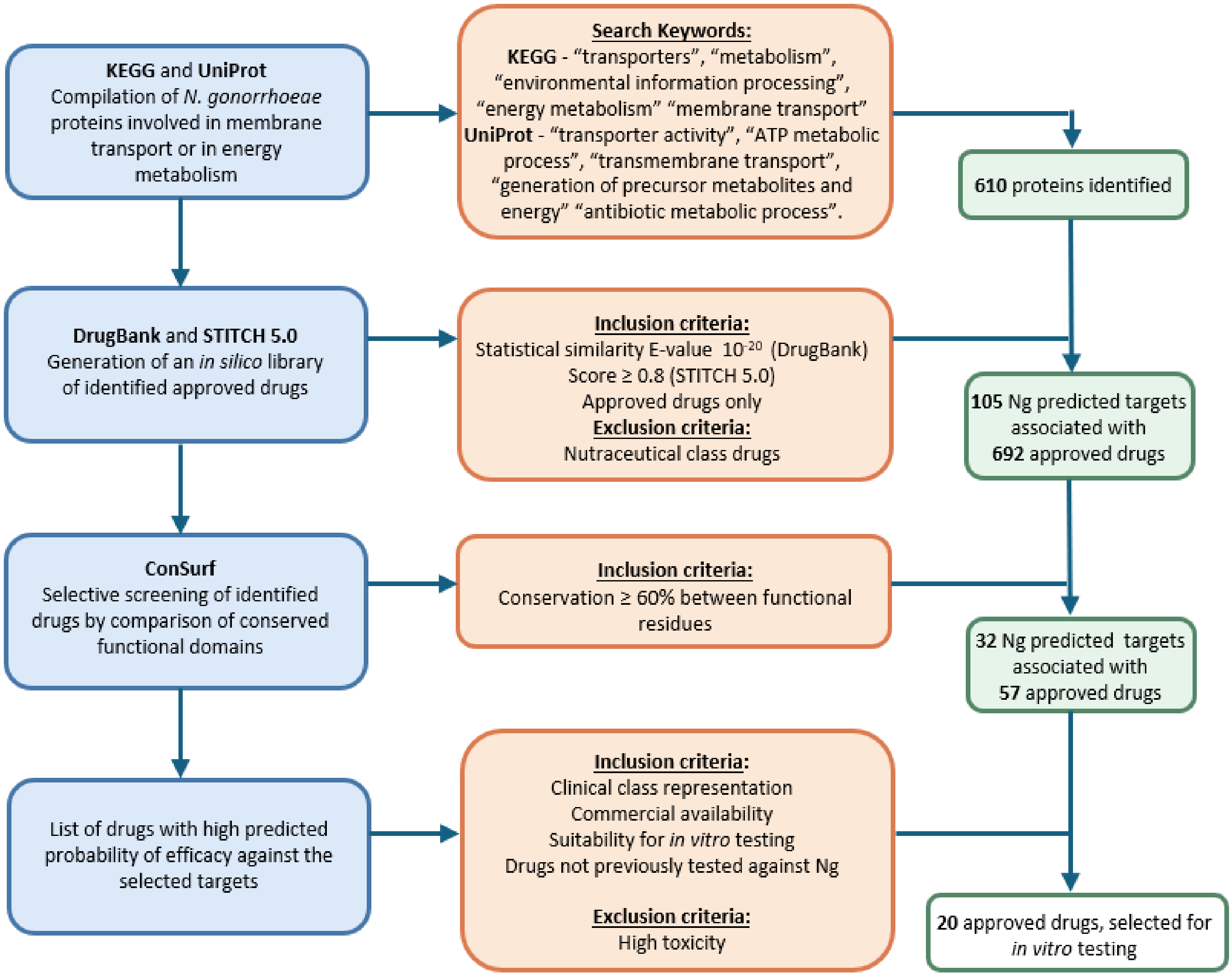

Figure S3. Flowchart illustrating the identification, curation, filtering and prioritisation process used to generate the final panel of approved drugs for *in vitro* testing against *N. gonorrhoeae* (Ng). The workflow includes retrieval of drug-target associations from DrugBank and STITCH 5.0, inclusion of approved drugs only, filtering based on functional residue conservation, and final prioritisation according to biological relevance, experimental feasibility and literature screening. Numbers indicate the drugs retained at each stage of the selection process.
